# Phylogenomic and biochemical analysis reassesses temperate marine yeast *Yarrowia lipolytica* NCIM 3590 to be *Yarrowia bubula*

**DOI:** 10.1038/s41598-021-83914-6

**Published:** 2021-03-09

**Authors:** Prashant Gaikwad, Swanand Joshi, Akshay Mandlecha, Ameeta RaviKumar

**Affiliations:** Institute of Bioinformatics and Biotechnology, Savitribai Phule Pune University, Ganeshkhind, Pune, Maharashtra 411 007 India

**Keywords:** Microbiology, Molecular biology

## Abstract

*Yarrowia* clade contains yeast species morphologically, ecologically, physiologically and genetically diverse in nature. *Yarrowia lipolytica* NCIM 3590 (NCIM 3590), a biotechnologically important strain, isolated from Scottish sea waters was reinvestigated for its phenotypic, biochemical, molecular and genomic properties as it exhibited characteristics unlike *Y. lipolytica*, namely, absence of extracellular lipolytic activity, growth at lower temperatures (less than 20 °C) and in high salt concentrations (10% NaCl). Molecular identification using ITS and D1/D2 sequences suggested NCIM 3590 to be 100% identical with reference strain *Yarrowia bubula* CBS 12934 rather than *Y. lipolytica* CBS 6124 (87% identity) while phylogenetic analysis revealed that it clustered with *Y. bubula* under a separate clade. Further, whole genome sequencing of NCIM 3590 was performed using Illumina NextSeq technology and the draft reported here. The overall genome relatedness values obtained by dDDH (94.1%), ANIb/ANIm (99.41/99.42%) and OrthoANI (99.47%) indicated proximity between NCIM 3590 and CBS 12934 as compared to the reference strain *Y. lipolytica*. No extracellular lipase activity could be detected in NCIM 3590 while *LIP2* gene TBLASTN analysis suggests a low 42% identity with e value 2 e^−77^ and 62% coverage. Hence molecular, phylogenetic, genomics, biochemical and microbial analyses suggests it belongs to *Yarrowia bubula*.

## Introduction

Yeasts from the *Yarrowia* clade differ distinctly from conventional yeasts and are widespread geographically in different ecological niches. Species within this clade are diverse and exist in both yeast and mycelial forms in order to adapt and colonize various environments. The most extensively studied and biotechnologically relevant species in the *Yarrowia* genus is *Yarrowia lipolytica* but other species have also been reported from different ecological niches such as animals, fresh/sea water and dairy. Species isolated from animal/insect related niche such as bacon, chicken and meat are *Yarrowia divulgata*^1^ from Denmark, USA and Hungary; *Yarrowia galli*^2^ (USA), *Yarrowia bubula* and *Yarrowia porcina*^3^ (Hungary), *Yarrowia* (*Candida*) *alimentaria*^4^ (Norway) while *Yarrowia yakushimensis*^5^ (Japan) and *Yarrowia parophonii*^6^ (Bulgaria) from insect guts. Species isolated from aquatic ecosystem, are *Yarrowia* (*Candida*) *phangngensis*^7^ (Thailand) and *Yarrowia keelungensis*^8^ (Taiwan). *Yarrowia* (*Candida*) *oslonensis*^4^ and *Yarrowia brassicae*^9^ were isolated from yogurt (Norway) and traditional Chinese sauerkraut, respectively. All the above mentioned species are non-fermentative and assimilate a wide variety of carbon sources^1–8^ suggesting phenotypic diversity and variability amongst them^10^. All the species in the *Yarrowia* clade are oleaginous and exhibit specific growth patterns while differing in their ability to accumulate the amount and type of lipid^11^. A number of lipases have been explored in the *Yarrowia* clade, the most prominent being extracellular lipase, Lip2p, encoded by the *LIP2* gene^12^. This gene is present in all the species of the *Yarrowia* clade but may not be expressed in active form in all the species^13^. Tolerance to NaCl (10%, w/v) differs among species with *Y. hollandica*, *Y. keelungensis*, *Y. divulgata*, *Y. porcina* and *Y. bubula* able to grow in high saline conditions^1,3,4,8^. Similarly, diversity in optimal growth temperature is observed, ranging from 21 °C for *Y. alimentaria* to 37 °C for *Y. phangngensis* with other *Yarrowia* species growing between 25 and 30 °C^11^. *Y. lipolytica* has often found to inhabit marine ecosystems including high saline waters^14,15^ as well as in contaminated milk, dairy products^16^, poultry^17^ and meat products^18^ in USA and Europe. This non-conventional strictly aerobic ascomycetous yeast generally regarded as safe (GRAS) by the American Food and Drug Administration FDA, USA has been exploited in several biotechnological, environmental and industrial applications. Some examples include heterologous host for production of pharmaceutically and industrially relevant proteins, enzymes, organic acids, biofuels, bioremediation of industrial and environmental wastes^19–22^ and is also considered as a very good model for dimorphism studies^23,24^. The strain NCIM 3590 obtained from National Collection of Industrial Microrganisms (NCIM), Pune, India is a psychotropic marine isolate from Scottish seawater^25^ and deposited in National Collection of Yeast Cultures as NCYC 789 and in Microbial Type Culture Collection, Chandigarh as MTCC 35. This strain has been studied for its potential in biodiesel production^26^, bioabsorption and bioremediation of heavy metals^27,28^, in nanoparticle synthesis and melanin production^29^. A survey of literature suggests that scarce information regarding its basic microbiology, growth and physiology exist and is the only *Y. lipolytica* strain reported to grow at lower temperatures (20 °C and less) and did not exhibit any extracellular lipase activity, a characteristic of *Y. lipolytica* strains. The classical identification of yeast species has traditionally been based on colony/cellular morphology and physiological characteristics with respect to their sugar assimilation, fermentation and oxidation capabilities^30^. These morphological and physiological characteristics are not sufficient to identify *Yarrowia* species as very often similar profiles between species are obtained. In this context, correct identification and classification of yeast is essential for basic biological and application based research and as the genomes of many type strains of species belonging to the *Yarrowia* clade have been sequenced, this information would be useful for identification. Hence, we re-examine this biotechnologically relevant strain using microbiological, biochemical, molecular biological and genome based studies.

## Results and discussion

### ITS and D1/D2 rDNA identification and phylogenetic analysis

**Figure 1:**
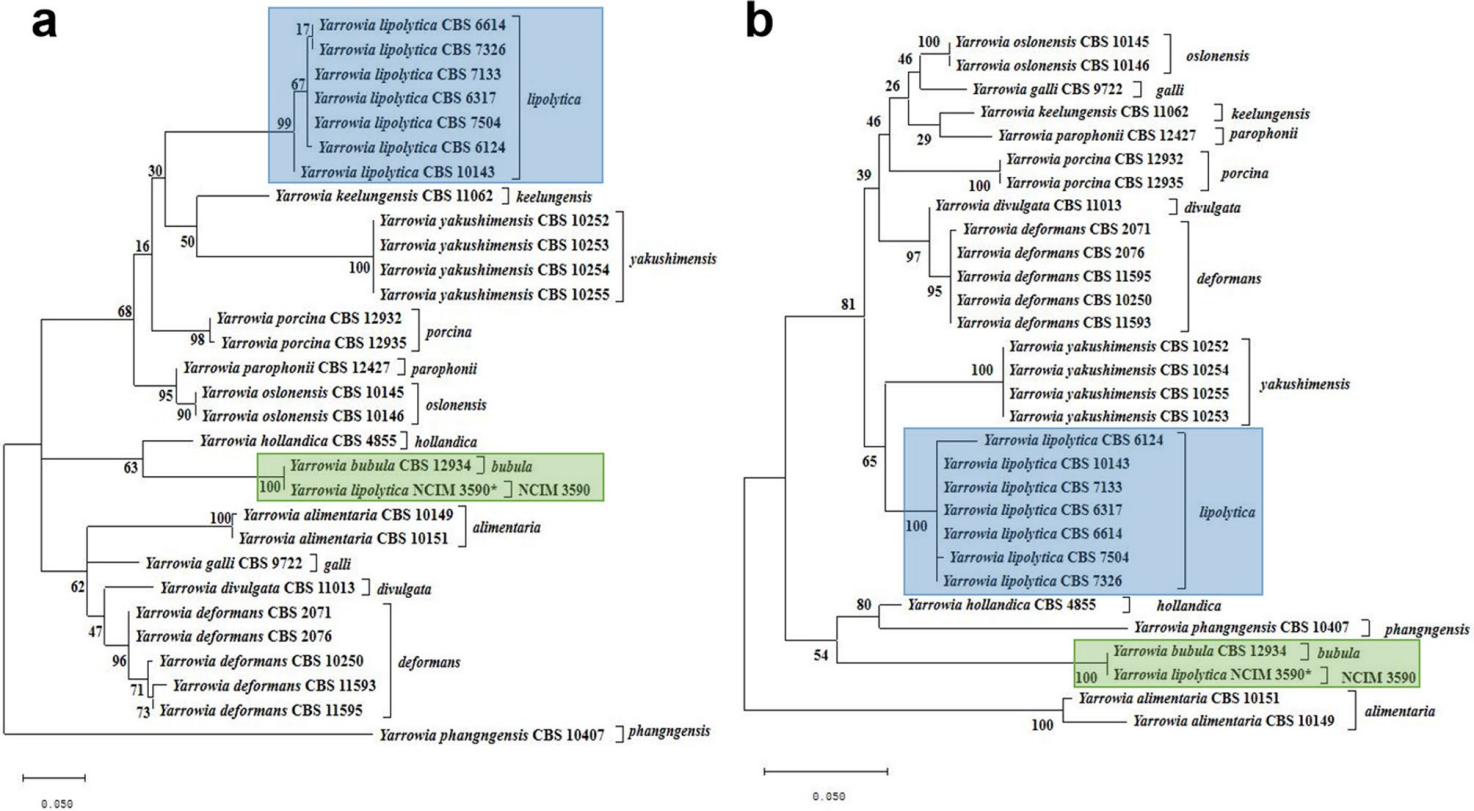
Phylogeny of NCIM3590. The phylogenetic tree analysis by maximum likelihood (ML) method showing the placement of NCIM 3590 and related species based on the analysis of (**a**) internal transcribed spacer (ITS) regions and (**b**) D1/D2 domains of the large subunit rRNA gene. The ML tree was obtained using the GTR + G + I model. The tree with the highest log likelihood is shown. The tree is drawn to scale with branch lengths measured in the number of base substitutions per site. All positions containing gaps and missing data were eliminated. Blue box represents *Y. lipolytica* as single clade/group and green as *Y. bubula* as single clade/group.

The sequenced lengths of ITS and D1/D2 regions of NCIM 3590 showed that it consisted of 311 and 446 nucleotides, respectively which have been submitted to GenBank (NCBI, USA) with accession numbers MK411246 and MK411222, respectively. BLAST analysis of ITS regions of NCIM 3590 showed 100% identity with the reference strain *Y. bubula* CBS 12934 (KY105958.1) with query coverage of 95%. The ITS sequence of NCIM 3590 when compared with *Y. bubula* CBS 12934 (consensus length 290 nt) showed 100% similarity index with gap number and gap length as 0 and divergence as 0%. In contrast, comparison with reference *Y. lipolytica* CBS 6124 (consensus length 311 nt), 83% similarity with gap number (5), gap length (11), divergence (14.2%) and sequence identity (87%) was seen. For D1/D2 sequences, NCIM 3590 showed 100% identity, query coverage, and sequence similarity with 0% divergence, gap length and gap number (0) with the reference *Y. bubula* CBS 12934 (NG_059943.1). Sequence comparison with *Y. lipolytica* CBS 6124, showed a similarity index of 89.5% with gap number and gap length (1), divergence (13%) and identity (87.5%). Generally, ITS region is selected as the standard fungal barcode for identification^31^ but, for yeasts it is recommended to use D1/D2 together with ITS region for identification and establishment of evolutionary relationships^32^. It has also been reported that strains showing more than 1% difference or with changes in more than 3 nucleotides in ITS and D1/D2 domains are likely to represent different species^4^. Thus, the molecular identification using ITS and D1/D2 sequences suggests that NCIM 3590 could belong to *Y. bubula*. Further the phylogenetic analysis of ITS and D1/D2 sequences from GenBank, NCBI showed that *Yarrowia* sp. could be separated into 13 clades (Fig. 1a,b and Table S1). All the seven different *Y. lipolytica* strains used in the study were grouped together in a single clade with 100% bootstrap support while the ITS and D1/D2 sequences of NCIM 3590 clustered with CBS 12934 suggesting its close relatedness. Since a close similarity between NCIM 3590 with *Y. bubula* and its distinct difference from *Y. lipolytica* strains was seen, the sequence comparisons of the strains from these clades/groups were analyzed and the results for ITS and D1/D2 given in supplementary information (Table S2). The ITS and D1/D2 genetic distances of NCIM 3590 with other *Y. lipolytica* species ranged from 0.392 to 0.407 and ~ 0.21 base substitutions per site, respectively (Table 1). The percent divergence over sequence pairs between NCIM 3590 with different *Yarrowia* groups was calculated using *p*-distance method and the results given in supplementary information (Table S4). NCIM 3590 was considered as a different group for comparison and establishing the evolutionary divergence. The base differences per site in ITS and D1/D2 sequences between each *Yarrowia* species was ascertained and averaged forming a dataset of 14 groups and divergence given (Table S4). The divergence for ITS and D1/D2 rDNA sequences with *Y. lipolytica* was 13.35 and 9.19%, respectively and no divergence seen with *Y. bubula* (Table S4). The variations found in their D1/D2 and ITS sequences are adequate to separate the *Yarrowia* species from one another^4,11^. Thus, as per delineation of species since less than 1% and no nucleotide difference was observed between NCIM 3590 and *Y. bubula* CBS 12934, the phylogenetic analysis suggests that they belong to the same species and is distinctly separated from the *Y. lipolytica* clade.

**Table 1:** Evolutionary distances between NCIM 3590 and various *Y. lipolytica* and *Y. bubula* strains using GTR + G + I model^a^.

Organisms	NCIM 3590 (base substitutions per site)	
	D1/D2	ITS
*Yarrowia lipolytica* CBS 6124	0.209	0.407
*Yarrowia lipolytica* CBS 6317	0.197	0.403
*Yarrowia lipolytica* CBS 6614	0.192	0.407
*Yarrowia lipolytica* CBS 7133	0.192	0.403
*Yarrowia lipolytica* CBS 7326	0.192	0.407
*Yarrowia lipolytica* CBS 7504	0.194	0.403
*Yarrowia lipolytica* CBS 10143	0.192	0.392
*Yarrowia bubula* CBS 12934	0.000	0.000
*Yarrowia lipolytica* CBS 6124	0.209	0.407
*Yarrowia lipolytica* CBS 6317	0.197	0.403

^a^Evolutionary distances between pairs of sequences are shown. Analyses were conducted using the General Time Reversible model. This analysis involved 30 nucleotide sequences There were a total of 403 positions (sites) for D1D2 and 314 (sites) for ITS in the final dataset. Evolutionary analyses were conducted in MEGA X.

### NGS and de novo genome assembly

In order to validate the above results, whole genome sequencing of NCIM 3590 was undertaken. The initial genome de novo assembly was carried out using SPAdes assembler (optimized with 10 million reads, *k*-mers: 21, 33, 55 and 77) enabling assembly of 2485 contigs representing with N50 of 39,245 bp (Table 2). Contigs (521) were found to be more than 10 kb length with an average contigs length of 8592 bp. The SPAdes^33^ assembler relies on “paired *de-Bruijn* graphs” (PDBG) approach which utilises a *k*-bimer based adjustment strategy for creating *de-Bruijn* graph using the paired-end reads. The genome is assembled using multiple *k*-mer sizes and eventually combining the reads into contigs. The assembler was initially designed for assembling prokaryotic genomes but later developed to accommodate large eukaryotic genomes. Assembled contigs were further scaffolded de novo using SSPACE that resulted in 2074 scaffolds with the N50 of 69,096 bp. SSPACE (SSAKE-based Scaffolding of Pre-Assembled Contigs after Extension) program is used to scaffold pre-assemblies produced by SPAdes. SSPACE^34^ requires paired-end data from next-generation sequencing technology, read orientation information, mean values and standard deviations of the insert sizes used in library preparation. Using paired-read sequencing data the assembler assess the order, distance and orientation of contigs and combines them into scaffolds. Based on the alignments the contigs are linked into scaffolds and N-characters (gaps) are placed between the connected scaffolds. As per assembly statistics, scaffolds (730) greater than 1 kb were considered for super-scaffolding/gap closing along with paired end data using SOAPdenovo2 with asm flags (3, 4) parameter (https://github.com/aquaskyline/SOAPdenovo2). SOAPdenovo2^35^ utilizes six modules namely, read error correction, *de Bruijn* graph (DBG) construction, contig assembly, paired-end (PE) reads mapping, scaffold construction, and gap closure. As a result of super-scaffolding method applied on greater than 1 kb scaffolds, the total number of scaffolds and non-ATGC count was decreased. The minimum scaffold length was increased from 1000 to 1032 bases (Table 2). The de novo genome assembly strategy was selected as an unbiased approach since it does not consider prior knowledge of the source DNA sequence length, layout or composition. Super-scaffolding and gap closure in the assembled scaffolds resulted in 485 super-scaffolds with N50/N90 values of 73,209/20,565 bp, respectively. The draft NCIM 3590 genome contains approximately 20,010,574 bp and has a GC content of 47%.

**Table 2:** Draft de novo genome assembly statistics of NCIM 3590.

	Contig	Scaffold	Draft genome
Contigs generated	2485	2074	485
Maximum contig length	277,850	406,748	406,748
Minimum contig length	500	500	1032
Average contig length	8592	10,333	41,258
Total contigs length	21,352,539	21,431,617	20,010,574
Total number of non-ATGC characters	346	35,429	33,863
Percentage of non-ATGC characters	0.002	0.165	0.169
Contigs ≥ 1 Kbp	1069	730	485
Contigs ≥ 10 Kbp	521	380	376
Contigs ≥ 1 Mbp	0	0	0
N50 value	39,245	69,096	73,209

The availability of completely annotated genome assembly is a significant advantage for the study of any organism. CLIB 122^36^ was the first genome reported to be fully sequenced and annotated. Additionally WSH-Z06, H222 (CLIB 80), W29 (CLIB 89), IBT 446 and PO1f. were fully sequenced and annotated at the chromosome level while few strains were assembled upto contigs and scaffolds level (https://www.ncbi.nlm.nih.gov/genome/browse#!/eukaryotes/yarrowia). The reference genomes, namely, *Y. bubula* CBS 12934^37^ and two *Y. lipolytica* strains CLIB 122and CLIB 89 (W29)^38^, were selected for comparison of whole genome sequencing data as these strains not only had a genome size similar to NCIM 3590 for assembly purpose and also the availability of complete genomic and annotation information.. In general, *Y. lipolytica* has a GC content reported to lie between 49 and 50%. The two reference strains CLIB 122 and CLIB 89 exhibited a GC content of 49.1 and 49.0% while for NCIM 3590 and *Y. bubula* CBS 12934 was 47 and 46.2%, respectively which was 2% lower than the *Y. lipolytica* strains (Table S5).

### Overall genome relatedness index (OGRI) and genome comparison

The digital DDH (dDDH) tools calculate inter-genomic distances using three formulas and convert these distances into percent-wise dDDH similarities. For distance calculation, Formula 1 utilizes HSP (High-scoring Segment Pairs) length and total lengths of the genome; Formula 2 uses identities and HSP length while Formula 3 uses identities and total lengths^39–41^. The calculated dDDH values for CBS 12934 with NCIM 3590 were found to be 98.8, 94.1 and 99.1% with Formulas 1, 2 and 3, respectively (Table 3) indicating that all three formulas suggest a high degree of similarity or relatedness. In contrast, the dDDH similarity values for NCIM 3590 were found to be ~ 17, 26 and 17% with Formulas 1, 2 and 3, respectively suggesting low level of relatedness. A dDDH similarity score of greater than or equal to 70% is a criterion for assigning two strains to the same species^40,42^ and hence the values obtained suggests that NCIM 3590 and *Y. bubula* CBS 12934 are related and belong to the same species. The distance between NCIM 3590 and *Y. bubula* CBS 12934 was 0.0209, 0.0077 and 0.0284 while between *Y. lipolytica* strains and NCIM 3590 was ~ 0.80, 0.169 and 0.83 with Formulas 1, 2 and 3, respectively (Table 3). Two genomes are considered to belong to the same species when they have a genome distance value (GD) of less than 0.0284^39,41^ and thus, based on the distance values also the two genomes of NCIM 3590 and *Y. bubula* CBS 12934 seems to be closely related. Another parameter for relatedness using dDDH, calculates the difference in G + C content (%) which was found to be 0.32% between NCIM 3590 and reference *Y. bubula* CBS 12934 while it was 1.94% and 1.87% for CLIB 122 and CLIB 89, respectively (Table 3). Meier-Kolthoff et al.^43^ suggested that a value greater than 1.0% indicates distinct species^43^. Since the difference in G + C content is greater than 1% between NCIM 3590and CLIB 122 and CLIB 89, it suggests that NCIM 3590 does not belong to the same species as CLIB 122 and 89*.*

**Table 3:** Genomic relatedness using dDDH and ANI of NCIM 3590 with CBS 12934 and with two reference strains CLIB 122 and CLIB 89.

Sr. no	Analysis	Formula/parameters	Query Genome	NCIM 3590	NCIM 3590	NCIM 3590
			Reference Genome	CBS12934	CLIB 89	CLIB 122
1	dDDH	Formula 1 (HSP length/total length)	DDH (%)	98.8	17	16.9
			Distance	0.209	0.8045	0.8051
			Prob DDH ≥ 70%	99.36	0	0
		Formula 2 (identities/HSP lengths)	DDH (%)	94.1	25.68	25.6
			Distance	0.0077	0.1698	0.1696
			Prob DDH ≥ 70%	96.7	0.01	0.01
		Formula 3 (identities/total lengths)	DDH (%)	99.1	17.1	17
			Distance	0.0284	0.8377	0.8382
			Prob DDH ≥ 70%	99.97	0	0
		Diff in GC%	–	0.32	1.87	1.94
2	ANI	ANIb (%)	–	99.41	80.02	80.04
		ANIm (%)	–	99.42	86.14	86.16
		OrthoANI (%)	–	99.47	80.25	80.18
	Interpretation			Same species	Distinct species	Distinct species

**Figure 2:**
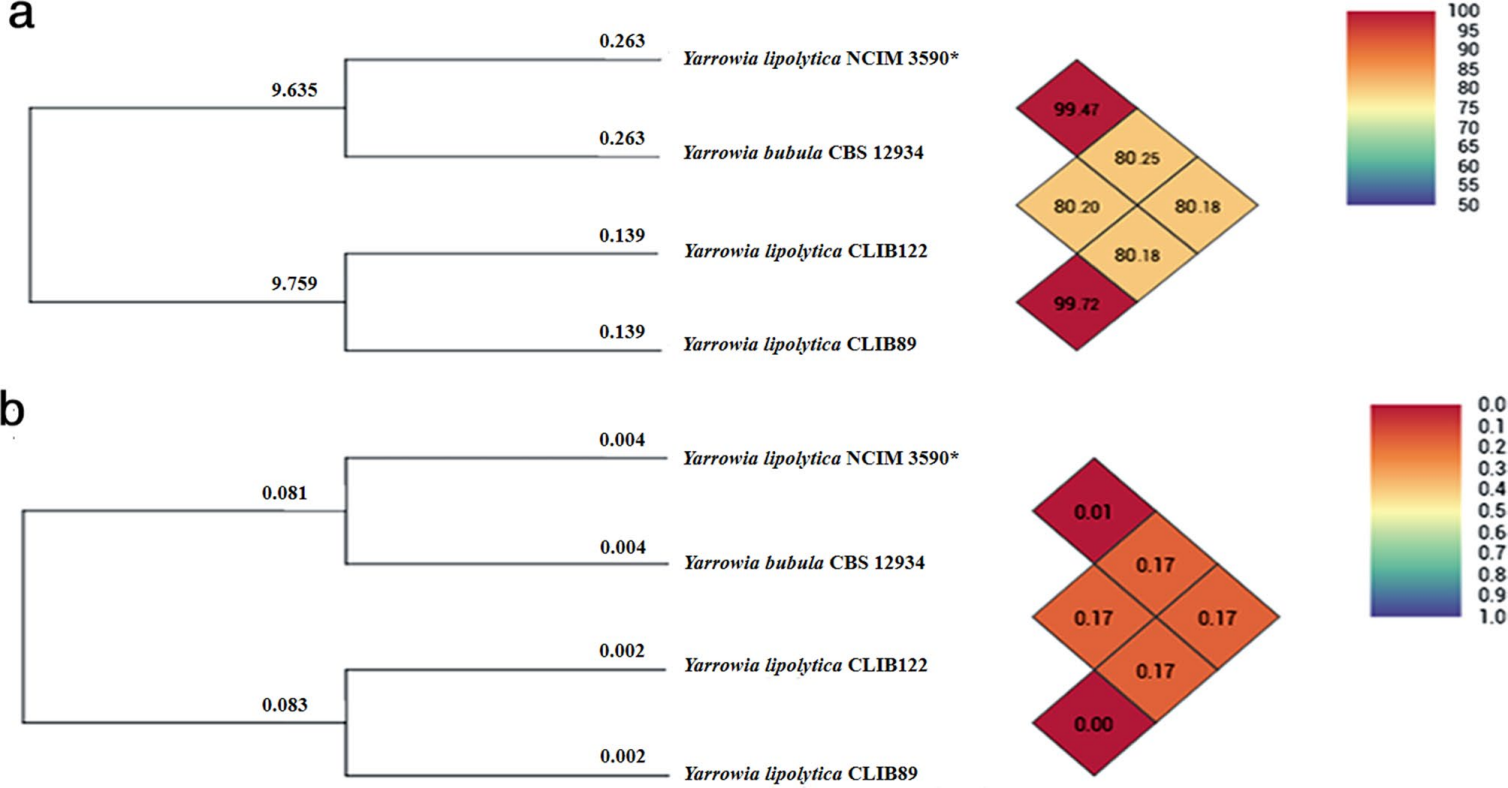
Dendrogram of the inter-species genomic relatedness for four *Yarrowia s*trains. Heat map generated using (**a**) Overall orthologous average nucleotide identity values (OrthoANI) and (**b**) GGDC distance among *Yarrowia* genomes was calculated using Orthologous Average Nucleotide Identity Tool (OAT) software version 0.90 *Y. lipolytica* CLIB 122 (GenBank accession GCA_000002525.1), *Y. lipolytica* CLIB 89 (W29) (GenBank accession GCA_001761485.1), NCIM 3590 (GenBank accession GCA_003571375.1) and *Y. bubula* CBS 12934 (GenBank accession GCA_900519075.1)**.** Values in color matrix boxes indicate the similarity percentage among the genomes.

Though the GGDC tool of dDDH has been effectively used for species delineation in prokaryotes, very few reports exist on its application in fungal and yeast systems. Mefteh et al.^44^ used this tool to show that the two strains of *Penicillium citrinum* genomes belong to the same species (DDH 97.3% and distance 0.004), the two strains of *Geotrichum candidum* are genetically distant (DDH 18.3% and distance 0.236) using Formula 2^44^. Similarly, relatedness between *Saccharomyces cerevisiae* S288c and four *Candida* species namely *C. auris* 6684, *C. albicans* (SC-5314 and WO-1), *C. lusitaniae* ATCC 42720 and *C. glabrata* CBS-138 has been studied^45^. Genomic relatedness was also determined using Average Nucleotide Identity (ANI) and OrthoANI (OANI) which are the mean of nucleotide identity values between the two organisms and have been widely used indices under OGRI^46^. The ANIb/ANIm/OANI values between CLIB 122 and CLIB 89 is 99.71%/99.70%/99.72% indicating that they belong to the same species (Fig. 2a). The ANIb/ANIm/OANI values between NCIM 3590 and CBS 12934 is ~ 99.41% while between CLIB 122 and CLIB 89 were ~ 80.00%, respectively (Table 3). The inter-genomic distance between NCIM 3590 and *Y. bubula* CBS 12934 was calculated to be 0.01 and between two *Y. lipolytica* species was 0, while between NCIM 3590 and *Y. lipolytica* species was 0.17 (Fig. 2b). Values greater than 96% and genome distance closer 0 indicate that strains belong to the same species^46^. The data obtained for NCIM 3590 and CBS 12934 using ANI, OrthoANI and dDDH suggests that both strains are genomically related to each other and corroborates with the established boundaries for genomic species delineation (95–96% for ANI and OANI, 70% for digital DDH)^46–48^. Our results suggest that the NCIM 3590 and *Y. bubula* CBS 12934 are closely related and belong to the same species.

Based on ITS and D1/D2 rDNA sequencing, whole genome comparison and OGRI, NCIM 3590 is likely to be *Y. bubula*. The strain has tremendous potential for biotechnological applications and scarce information with respect to its basic microbiological aspects is available and hence, it was deemed necessary to investigate these aspects.

### Colony morphology

As no information regarding phenotypic characterization, namely, size, form, elevation, margin edge, surface, opacity and chromogenesis of NCIM 3590 is available in literature, the yeast growth on different culture media was undertaken^49^. The marine yeast NCIM 3590 was able to grow on all the eight different growth media studied (Fig. S1; Table S6), and compared with the reference strains *Y. lipolytica* CBS 6124^14^ and *Y. bubula* CBS 12934^3^ (Table 4). Of these, five media (MEA, MGYP, YPG, PDA and TA) exhibited similar colony characteristics and were opaque, white in colour, circular, 2–5 mm in diameter, entire margins and edge, umbonate, dome-shaped elevation with shiny, wrinkled surface. In contrast, the reference strain when grown on MEA (5%, w/v) for 72 h showed tannish-white butyrous colonies^14^ whereas *Y. bubula* showed cream coloured butyrous colonies^3^.

**Table 4:** Comparison of NCIM 3590 with different *Yarrowia* sp.

Sr. no	Organism	Colony	Cellular morphology/Budding	Temperature (°C)	NaCl	Sugar assimilation	GC %	References
	Type strain							
01	*Yarrowia lipolytica*	Butyrous, hyphal, tannish white	Spherical, ellipsoidal/multilateral	25	–	NAG +, Man +, Galv, VFM −	48.98	11
	CBS 6124							
02	*Yarrowia divulgata*	Butyrous, smooth, cream	Spherical, ellipsoidal/polar	25.30	+	NAG +, Man +, Gal −, VFM −	50.20	1
	CBS 11013							
03	*Yarrowia *(*Candida*)* galli*	Butyrous, cream, hirsute	Spherical, ellipsoidal/multilateral	30	+	NAG −, Man +, Gal +, VFM +	49.10	2
	CBS 9722							
04	*Yarrowia keelungensis*	Smooth, entire, tannish white	Ellipsoid to elongate/multilateral	25, 30, 35	+	NAG +, Man +, Gal +, VFM −	48.10	8
	CBS 11062							
05	*Yarrowia *(*Candida*)* phangngensis*	Butyrous, cream, hirsute	Spherical, ellipsoidal/multilateral	25, 30, 35, 37	+	NAG −, Man +, Gal −,	43.30	7
	CBS 10407							
06	*Yarrowia *(*Candida*)* oslonensis*	Not reported	Ovoid to globose/multilateral	25, 27, 30	–	NAG +, Man −, Gal +, VFM −	50.60	4
	CBS 10146							
07	*Yarrowia *(*Candida*)* hollandica*	Not reported	Ovoid to globose/multilateral	25, 27, 30	+	NAG +, Man +, Gal +	47.80	4
	CBS 4855							
08	*Yarrowia *(*Candida*)* alimentaria*	Not reported	Ovoid to globose/multilateral	21, 25, 27	–	NAG +, Man −, Gal +, VFM −	49.20	4
	CBS 10151							
09	*Yarrowia bubula*	Butyrous, smooth, cream	Ellipsoid/multilateral	25	+	NAG +, Man +, Gal +, VFM −	46.20	3
	CBS 12934							
10	*Yarrowia porcina*	Butyrous, smooth, cream	Ellipsoid/multilateral	25, 30	+	NAG +, Man +, Gal +, VFM −	43.70	3
	CBS 12935							
11	*Yarrowia yakushimensis*	Butyrous cream	Ovoid to elongate/multilateral	25	ND	NAG −, Man +, Gal −, VFMnd	48.30	5
	CBS 10254	Undulate margin						
12	*Yarrowia deformans*	Mucoid, cream , wrinkled	Ellipsoid, elongate/multilateral	25	ND	NAG +, Man +, Gal −, VFM −	49.50	5
	CBS 2071							
13	*Yarrowia parophonni*	Butyrous, smooth, cream	Ovoid to globose/multilateral	25	ND	NAG +, Man +, Gal + ,	ND	6
	CBS 12427							
14	*Yarrowia lipolytica*	Umbonate, Opaque, wrinkled	Spherical, ellipsoidal/bilateral	10, 15, 20, 25, 28	+	NAG +, Man +, Gal +, VFMnd	47.00	Present study
	NCIM 3590							

(+) Positive, (−) Negative, *v* Variable, *ND* not determined, *NAG* *N*-Acetyl-d-glucosamine, *Man* d-Mannitol, *Gal* d-galactose, *VFM* vitamin free medium; GC%—https://www.ncbi.nlm.nih.gov/genome/browse#!/eukaryotes/yarrowia.

*CBS* Central Bureau voorSchimmelcultures, Utrecht, The Netherlands, *NCIM* National Collection of Industrial Microorganisms, Pune, India.

The yeast when grown on YLDM, YES and YPO showed varied colony morphologies (Table S6). YLDM is a selective medium used to differentiate *Y. lipolytica* from other yeasts as its colonies produce a unique deep brown colour after 24 h due to the presence of pigments^49,50^. On YLDM, NCIM 3590 showed circular, white coloured, hat-shaped colonies (Fig S1f) in contrast to the reference *Y. lipolytica* strain. On YES, media, no growth was seen when grown on minimal medium YNB-sucrose (1%, w/v) suggesting its inability to utilize the sugar while, it was able to assimilate sucrose when grown on complete medium, with white wrinkled surface, erose margin and fuzzy growth (Fig. S1g). YES agar, is routinely used for sporulation tests in yeasts^3^. No sporulation was seen in NCIM 3590 upto 15 days. Most *Yarrowia* strains are haploid (the only known exception is (CBS 6124) and therefore cannot sporulate unless being mated. On YPO, the colony morphology is given in Table S6 and no zone of clearance was observed in 72 h suggesting the absence of extracellular lipolytic activity (Fig. S1h). This is unlike *Y. lipolytica* which is a known producer of extracellular lipase and shows a clear zone of clearance when grown on YPO. Hence, growth patterns for NCIM 3590 were found to be different from the reference *Y. lipolytica* strain.

### Cellular morphology

**Figure 3:**
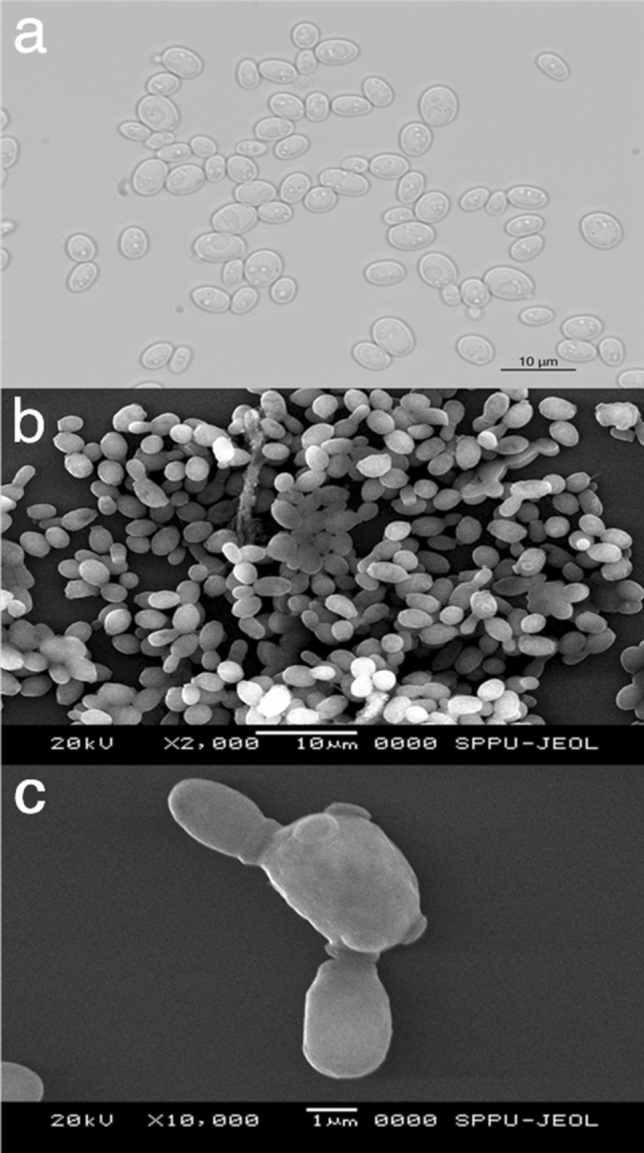
Cell morphology of NCIM 3590. (**a**) Light microscopy and (**b**) SEM and micrographs of NCIM 3590 strain grown on YNBG broth for 72 h. Micrographs showing Bar 10 µm. (**c**) shows budding and the arrows indicates bud scar. Bar 1 µm.

The light microscopic image (Fig. 3a) and scanning electron micrographs (Fig. 3b,c) of cells grown for 72 h in YNBG liquid media were spherical to ellipsoidal, 3–6 µm in size and displayed unipolar or bipolar budding. Elongated yeast forms were observed after 5 days of incubation and no filaments were observed even when grown for more than 7 days. *Yarrowia* sp. are dimorphic exhibiting yeast cells, pseudo-hyphae and hyphae depending on growth conditions.

A comparison of the cellular morphology and budding pattern between different *Yarrowia* sp. is given in Table 4. It is to be noted that while most of *Yarrowia* sp. reported so far show multilateral budding, NCIM 3590 shows bilateral budding pattern. In YNB medium, *Y. lipolytica* grows as the yeast form with a polar budding while hyphal growth can be induced either by *N*-acetyl-d-glucosamine (NAG) or by adding serum to the culture medium^51^. No growth of NCIM 3590 upto 5 days could be seen in vitamin free media and serum (1%, v/v). In NAG (1%, w/v) and serum (10%, v/v) the cells grew in yeast form and no transition to the filamentous form was observed. This is in agreement with the earlier report by Bankar et al.^25^ wherein, only yeast forms were observed for NCIM 3590^24^.

### Effect of temperature and salt on growth of NCIM 3590

**Figure 4:**
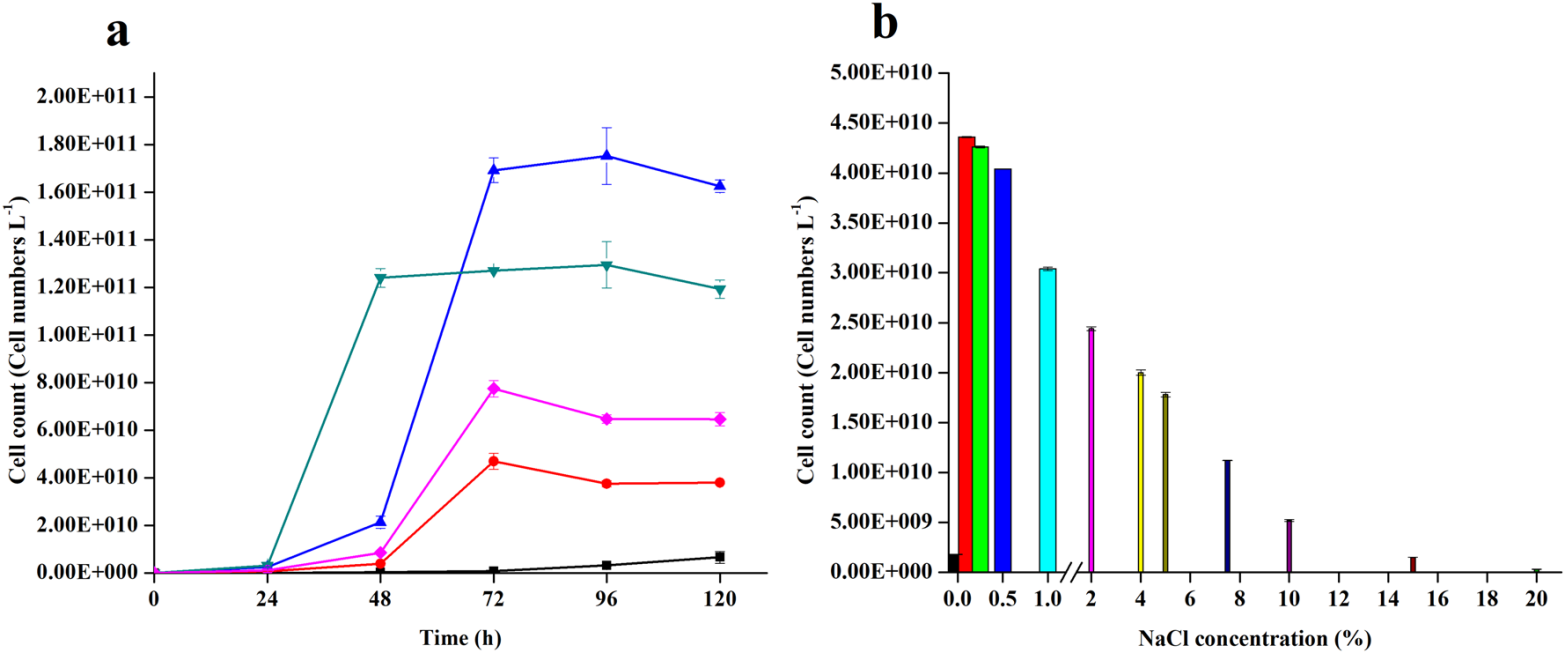
Effect of temperature and salt on growth of NCIM 3590. (**a**) Effect of temperature on the growth of NCIM 3590. The yeast was grown on YNBG at different temperatures for 120 h. Temperature symbols—10 °C (black filled square), 15 °C (red filled circle), 20 °C (blue triangle), 25 °C (dark cyan inverted triangle), 28 °C (magenta filled square). Data points are mean values from triplicate experiments with their standard deviation. (**b**) Effect of sodium chloride concentration on growth of NCIM 3590. Salt legends – 0.0% (black filled square), 0.1 (red filled square), 0.25% (green filled square), 0.5% (blue filled square), 1% (cyan filled square), 2% (magenta filled square), 4% (yellow filled square), 5% (dark yellow filled square), 7.5% (navy filled square), 10.0% (purple filled square), 15.0% (wine filled square), 20.0% (olive filled square). All experiments were carried out in duplicates and the mean values were recorded and the standard deviations (n = 2) are indicated as error bars. Different coloured bar indicates different concentration of NaCl used.

The effect of different temperatures and salt concentrations on growth of NCIM 3590 strain was evaluated. The yeast grew at temperatures between 10 and 28 °C with optimal growth at 20 °C and no growth on YNBG was observed at 30 °C and beyond as shown in Fig. 4a. Being a marine isolate, the effect of salt was studied on the yeast NCIM 3590 upto 72 h (Fig. 4b). The yeast was able to grow well upto 10% NaCl/5% glucose. Lesser growth was seen at higher salt concentration (15 and 20% NaCl/5% glucose) after 120 h.

On comparison with other *Yarrowia* sp. (Table 4), diversity in growth temperature and salt concentration is seen. In general, most of the *Yarrowia* sp. reported to date grow optimally between 25 and 30 °C. For example, *Y. lipolytica* CBS 6124^14^*, Y. divulgata* CBS 11013^1^*, Y. galli* CBS 9722^2^*, Y. oslonensis* CBS 10146^4^ and *Y. hollandica* CBS 4855^4^ grew upto 30 °C. Growth of *Y. porcina* CBS 12935^3^ and *Y. keelungensis* CBS 11062^8^ is reported upto 35 °C while *Y. phangngensis* CBS 10407^7^ could grow upto 37 °C. As of date, *Y. alimentaria* CBS 10151^4^ is the only reported strain able to grow between 15 and 25 °C with an optimal growth at 21 °C^11^ while NCIM 3590 was the only *Yarrowia* strain with an optimal growth temperature of 20 °C. Amongst the *Yarrowia* clade, strains reported to grow in 10% NaCl were *Y. divulgata* CBS 11013^1^*, Y. galli* CBS 9722^2^*, Y. bubula* CBS 12934^3^*, Y. porcina* CBS 12935^3^*, Y. hollandica* CBS 4855^4^*, Y. phangngnensis* CBS 10407^7^ and *Y. keelungensis* CBS 11062^8^ while *Y. lipolytica* CBS 6124^14^*, Y. keelungensis* CBS 11062^8^ and *Y. alimentaria* CBS 10151^4^ were unable to grow at this concentrations. Thus, a variation amongst *Yarrowia* species was observed as they could have adapted to the diverse ecological niches they were isolated from. Hence, based on growth at low temperature and high salt concentrations, it suggests that NCIM 3590 clearly differs phenotypically from *Y. lipolytica* CBS 6124.

### Biochemical characterization

#### Sugar assimilation studies

The assimilation and fermentation studies of NCIM 3590 were carried out on differing sugars and sugar alcohols (Table 4). The test for glucose fermentation was found to be negative as no gas production could be detected which corroborates the results obtained with other *Yarrowia* sp.^1–8^. Assimilation test for NCIM 3590 at 72 h were analysed using the metabolic fingerprinting database in Biolog. Carbon compounds strongly assimilated by NCIM 3590 were d-glucose, i-erythritol, d-gluconic acid, 2-keto d-gluconic acid and *N*-acetyl-d-glucosamine (NAG). Weak assimilation was seen for d-galactose, salicin, l-sorbose, d-xylose, l-arabinose, d-arabinose, d-ribose, glycerol, d-mannitol, succinate while no assimilation could be seen for inulin, sucrose, d-raffinose, d-mellibiose, d-trehalose, maltose, α-Methy l-d-glucoside, d-cellobiose, l-rhamnose and d-glucosamine (Table S7). The results from Biolog suggests NCIM 3590 as *Y. lipolytica* with probability score 1.0, similarity value (SIM) of 0.896 and distance of 1.543. A species to be considered acceptable for identification must have a distance value greater than 5.0 and a SIM of greater than 0·75. Based on the probability score and SIM value NCIM 3590 can be identified as *Y. lipolytica*. However, the distance value is much lower than the acceptable value of greater than 5. Also to be noted that the Biolog database has only *Y. lipolytica* and no other *Yarrowia* species listed in it and hence the best hit amongst the available database with *Y. lipolytica* is seen.

Sugar assimilation and utilization patterns also illustrate the diversity and adaptation amongst strains thereby, offering a convenient key for yeast identification. Upon comparison (Table 4), all *Yarrowia* species assimilate glucose, glycerol and i-erythritol while strains with differential assimilation of sugars such as NAG, d-mannitol and d-galactose have been reported. NAG could not be assimilated by *Y. galli* CBS 9722^2^, *Y. yakushimensis* CBS 10254^5^ and *Y. phangngensis* CBS 10407^7^. d-mannitol assimilation was not observed in *Y. alimentaria* CBS 10151^4^ and *Y. oslonensis* CBS 10146^4^ while d-galactose could not be utilized by *Y. divulgata* CBS 11013^1^, *Y. yakushimensis* CBS 10254^5^, *Y. deformans* CBS 2071^5^ and *Y. phangngensis* CBS 10407^7^. However, all the three sugars could be assimilated by NCIM 3590, suggesting that it behaved differently in its sugar assimilation profile. The three strains *Y. porcina* CBS 12935^3^, *Y. hollandica* CBS 4855^4^ and *Y. keelungensis* CBS 11062^8^ are reported to grow at 30 °C and above while NCIM 3590 is known to grow at lower temperature. As of date *Y. alimentaria* CBS 10151^4^ is the only reported strain able to grow at lower temperature^11^ but its inability to assimilate mannitol differentiates it from NCIM 3590.

#### Extracellular lipase activity

*Yarrowia lipolytica* is a known producer of high amounts of extracellular lipase and hence NCIM 3590 was screened for lipolytic activity by culturing on TO and YPO at 20 °C for 48 h with no clearance zone observed after 96 h incubation. The extracellular lipolytic activity in NCIM 3590 was also investigated on different broth media namely, YNBG, YPG, YPGO, YPGTr and YPGTw. Negligible amount of extracellular lipase activity was detected in supernatant of YNBG, YPG, YPGO, YPGTw under the given assay conditions while good cell growth was seen after 72 h incubation at 20 °C (Table S8). *Y. lipolytica* PO1d, a known producer of extracellular lipase produced 20 and 50 U/mL of lipase activity in YNBO (Yeast nitrogen base containing 1% (w/v) olive oil) and YPGO, respectively while *Y. lipolytica* strains, ATCC20460 and IMUFRJ 50682, produced up to 30 U/L on unsupplemented olive mill wastewater (OMW)^52^. *Y. lipolytica* CECT 1240 (ATCC 18942)^53^ and *Y. lipolytica* W29 (ATCC 20255)^54^ showed higher lipolytic activity of 700 and 770 U/L with YNBOandYPDO*.*

Thus, though variability in the levels of extracellular levels of lipase are seen in *Y.lipolytica*, all strains produced it unlike as that seen in case of NCIM 3590 which did not show any significant levels of activity.

#### *LIP2* gene analysis

As no significant extracellular lipase activity was detected in the crude supernatant, bioinformatic analysis was carried out to determine the presence of *LIP2* gene in NCIM 3590. Extracellular lipase, Lip2, encoded by *LIP2* gene (Gene Id YALI0A20350g) is a 334-amino acid precursor protein containing the putative 13-amino acid signal sequence^13^. The TBLASTN of *LIP2* gene showed 42% identity and 60% coverage with e value 2 e^−77^ with scaffold 57 of NCIM 3590 (Table S9). Further, the matched coordinates 100,268 to 101,158 from scaffold 57 (NKYT01000426.1) were used to identify the ORFs using the NCBI ORF finder. The generated 8 ORFs were used as query to carry out BLASTP (Reverse BLAST) against the *LIP2* gene. Out of 8 ORFs generated only one ORF showed 44% identity (113/254 aa) with e value 9 e^−79^ and 62% coverage (158/254 aa). To validate the results, a similar TBLASTN for *LIP2* gene was carried out with reference CLIB 122 and CLIB 89 which showed 100% identity and coverage while in reverse blast, one ORF out of 9, resulted in 100% identity and coverage with *LIP2*. Thus it seems that the ORF obtained from NCIM 3590 shows low homology to *LIP2*. According to Meunchan et al.^14^, while *LIP2* gene is likely to be present in all members of the clade it has been suggested that the gene has undergone a number of evolutionary events with a high number of duplications. Differing degrees of homologies amongst them exist and 11 lipases homologous to YlLip2 seen of which many were found to be transcriptionally inactive while others were actively expressed as in *Y. lipolytica*, *Y. galli* and *Y. phangngensis*^13^*.* Hence, low e value, percent identity and coverage in case of NCIM 3590 suggests that the lipase from NCIM 3590 may be transcriptionally inactive or may not belong to the *LIP2* family.

In conclusion, this study reassesses the strain NCIM 3590 based on molecular, phylogenetic, genomic, biochemical and microbiological data. Based on this, we suggest that NCIM 3590 and *Y. bubula* CBS 12934 belong to same species. The availability of NCIM 3590 genome will help in providing a platform for elucidating its potential applications and contribute to the understanding of this unusual *Yarrowia* strain with optimum growth temperature at 20 °C.

## Materials and methods

### Media and culture conditions

NCIM 3590 obtained from National Collection of Industrial Microorganisms (NCIM), NCL, Pune, India was maintained on Yeast extract Peptone Dextrose agar (YPD; Yeast extract, 0.3%; Peptone, 0.5%; Dextrose, 1%) at 20 °C for 48 h.

### ITS and D1/D2 sequencing and phylogenetic analysis

Colonies of NCIM 3590 were suspended in saline and genomic DNA was isolated using geneO-spin Microbial DNA isolation kit (geneOmbio Technologies, Pune, India) in accordance with the manufacturers instructions. The Internal transcribed spacer, ITS1 (5′TCCGTAGGTGAACCTGCGG-3′) and ITS4 (5′-TCCTCCGCTTATTGATATGC-3′)^55^ and for the D1/D2 domain, primers NL-1 (5′-GCATATCAATAAGCGGAGGAAAAG-3′) and NL-4 (5′-GGTCCGTGTTTCAAGAGG-3′)^56^ were used and amplified using standard PCR reaction with an annealing temperature of 54 and 55 °C, respectively. The products were purified by using a ExoSAP-IT PCR product Purification kit (Invitrogen Bioservices, India) and sequenced using an ABI PRISM Big Dye Terminator V3.1 kit (Applied Biosystems, USA) on 3130 Genetic analyser Automated DNA sequencing machine. The sequences were analyzed using Sequencing Analysis 5.2, ChromasPro v3.1 and BLAST analysis performed using BLASTN at NCBI server (http://www.ncbi.nlm.nih.gov/BLAST).

Phylogenetic analysis was performed with ITS and D1/D2 sequences of different *Yarrowia* species taken from NCBI database. All sequences were aligned separately using Clustal W^57^ with default parameters in MEGA X^58^ (Molecular Evolutionary Genetics Analysis) software. For both ITS and D1/D2 rDNA set the best fit nucleotide substitution model was determined using Maximum likelihood (ML) criterion. ML tree was constructed using general time-reversible model with gamma-distributed rates of variation among sites and a proportion of invariable sites (GTR + G + I model). The reliability of the trees was tested by bootstrapping with 1000 replicates. The tree is drawn to scale with branch lengths in the same units as those of evolutionary distances (number of base substitutions per site) which is used to infer the phylogenetic tree. Percent divergence at nucleotide level was calculated using *p*-distance method in MEGA X (www.megasoftware.net).

### NGS and de novo genome assembly

The genomic DNA from NCIM 3590 was extracted by CTAB method followed by NEXTFlex DNA library preparation and Illumina NextSeq 500 paired-end sequencing according to the manufacturer’s instructions. In total, ~ 41 Million paired-end reads (150 bp) were generated (estimated coverage ~ 600 ×). The generated paired-end reads of 150 bps were processed further for de novo genome assembly. Quality control of reads was checked with FastQC v2.2 (http://www.bioinformatics.babraham.ac.uk/projects/fastqc/) and trimming carried out using an in-house perl script. Reads were assembled into contigs using SPAdes v3.1^33^ and were further assembled into scaffolds with SSPACE^34^. Scaffolds were checked for the length and the scaffolds which are above 1000 bases length were used for super-scaffolds and gap closure using SOAPdenovo2 tool^35^. The reads obtained were submitted to Sequence Read Archive (SRA) and scaffolds to Genbank in NCBI repository.

### Overall genome relatedness index (OGRI) and genome comparison

The OGRI between genome of NCIM 3590 and the reference genomes of CLIB 122, W29 (CLIB 89) and CBS 12934 was determined using digital DNA-DNA hybridization (dDDH) and Average Nucleotide Identity (ANI). The dDDH was calculated using the web-based DSMZ service (http://ggdc.dsmz.de) available with Genome-to-Genome Distance Calculator (GGDC 2.0) with BLAST method. The ANI was determined using JspeciesWS^48^ while orthoANI was calculated using Orthologous Average Nucleotide Identity Tool^46^ (OAT).

### Colony morphology of NCIM 3590

The colony morphology was studied by growing the yeast on different media as described by Kurtzman et al.^49^. The strain was grown for 96 h at 20 °C on different agar media (w/v) namely; (a) Malt extract agar (ME), (b) Malt extract Glucose Yeast extract Peptone agar (MGYP), (c) Potato Dextrose agar (PD), (d) Yeast extract Peptone Glucose agar (YPG), (e) Tributyrin agar (T), (f) *Yarrowia lipolytica* Differential Medium (YLDM), (g) Yeast extract Sucrose agar (YES) and (h) Yeast extract peptone olive oil agar (YPO). Colony morphology of the strain was ascertained microscopically by using trinocular microscope (Model no. NSZ-606) Lawrence and Mayo, London. Media composition details given in supplementary information.

### Microscopy studies

Bright field and Scanning Electron Microscopy (SEM) were carried out to determine the morphology. Wet mounts of the washed *Yarrowia* cells grown for 72 h under shaking conditions (120 rpm) at 20 °C in Yeast Nitrogen base containing Glucose (1%, w/v) (YNBG) were prepared. Cell morphology was ascertained on a slide by use of Axioskope 40 light microscope attached with a photographic equipment and images acquired at 100 × magnification (ProgRes Capture Pro 2.7 and the software AxioVison Rel. 4.8)^59^.

For SEM, the separated cells were fixed in a 3% glutaraldehyde solution in 0.1 M Phosphate Buffered Saline (pH 7.2) for 30 min and washed three times in the same buffer. After fixation the cells were dehydrated in a series of alcohols of increasing concentrations (35, 50, 75, 95% and absolute ethanol) for 20 min at each stage and then with Hexamethyldisilazane for 10 min. The specimens were dried overnight on cleaned grease free silicon wafer and sputtered with a platinum–carbon mixture in JFC-1600 (JEOL, Japan) vacuum evaporator and observed under the SEM (JEOL JSM-6360A)^60^.

#### Effect of temperature and salt on growth of NCIM 3590

For pre-inoculum, the yeast cells were grown in liquid YNBG and incubated on a rotary shaker (120 rpm) at 20 °C for 48 h, cells harvested and washed twice with autoclaved distilled water and centrifuged at 10,000*g* for 10 min. The cell pellet was re-suspended in water and 1 OD cells (1 OD ~ 2 × 10^7^ CFU/mL) inoculated per 100 mL media as mentioned below. To study the effect of temperature on growth of NCIM 3590, batch experiments were carried out. Cells (0.5 OD per 50 mL media) were inoculated into YNBG and incubated at different temperatures (5, 10, 15, 20, 25, 28, 30 and 35 °C) on a rotary shaker (120 rpm) for varying time intervals (24–120 h) and growth assessed every 24 h. The cells were spun at 10,000*g* for 10 min at 4 °C and the pellet was washed twice with sterile distilled water, vortex mixed to separate the cells, OD_600_ taken and cell dry weight determined by freeze-drying the cells. Similarly, to study the effect of salt, different concentration of sodium chloride (0, 0.1, 0.25, 0.5, 1, 2, 4, 5, 7.5, 10, 15 and 20%, w/v NaCl) in 5% glucose on NCIM 3590 growth was determined as mentioned above.

### Sugar assimilation

The sugar assimilation profile of NCIM 3590 was determined using Biolog system (Biolog MicroStation with Microlog System, Release 4.20, Biolog, Hayward, CA, USA, (http://www.biolog.com/microID.html). Culture was grown on YPD agar for 72 h at 20 °C and the yeast suspension was prepared in 15 mL sterile water at inoculum density transmittance level 47 ± 2% (corresponds to 0.33 Optical density (OD_600_ nm) or 6 × 10^6^ colony forming units per mL (CFU/mL). The Biolog YT MicroPlate was inoculated with 100 µL of cell suspension and incubated for 24, 48, 72 and 96 h at 20 °C. Colorimetric change in each well was referenced against control wells and scored as mentioned in the protocol.

The fermentation test was carried out manually by Kurtzman et al.^49^ wherein 0.1 OD cells were inoculated in YNB containing 1% (w/v) glucose in medium size tubes containing inverted durhams tube^49^. Bromothymol blue was used as an indicator which on acid production changes the medium color from blue to green or yellow. Gas production is evidenced visually by presence of visible air bubbles trapped inside the Durham tube.

### Lipase activity

Pre-inoculum was carried out as above and washed cell pellet (0.5 OD per 50 mL) was inoculated into different media and incubated at 20 °C for 72 h. The media used were as follows: (a) Yeast Nitrogen Base containing Glucose (YNBG), (b) Yeast extract Peptone Glucose; (YPG), (c) Yeast extract Peptone Glucose Olive oil (YPGO), (d) Yeast extract Peptone Glucose Tributyrin (YPGTr) and (e) Yeast extract Peptone Glucose Tween 80 (YPGTw). Stock solutions of fatty acid (10% olive oil and 10% tributyrin) were subjected to sonication three times for 1 min each on ice, autoclaved separately and added into liquid media buffered with 50 mM phosphate buffer, pH 6.8. The samples were removed after 72 h to determine lipase activity, soluble protein content and cell wet weight. The cells were harvested by centrifugation at 5000*g* for 10 min at 4 °C, the supernatant obtained was used as extracellular lipase source. Spectrophotometric method using *p*-nitrophenyl palmitate (*p*-NPP) as a substrate was used with slight modification for measurement of lipase activity^12,61^. Protein concentration was estimated by the method of BCA with bovine serum albumin as a standard^62^. The media composition, lipase enzyme assay have been provided in supplementary material.

### Lipase gene (*LIP2*) analysis

*Yarrowia lipolytica *(YALI) Lip2 protein sequence (YALI0A20350g) was used as query for TBLASTN on the 14 different *Yarrowia* species namely CBS 10151 (GCA_900518985.1), CBS 12934 (GCA_900519075.1), CBS 2071 (GCA_900519085.1), CBS 11013 (GCA_900519045.1), CBS 9722 (GCA_900519055.1), CBS 4855 (GCA_900519065.1), CBS 11062 (GCA_900519035.1), CLIB 89 (GCA_001761485.1), CLIB 122 (GCA_000002525.1), NCIM 3590 (GCA_003571375.1), CBS 10146 (GCA_900519015.1), CBS 10407 (GCA_900519005.1), CBS 10407 (GCA_900519025.1) and CBS 10253 (GCA_900518995.1). The best hit of NCIM 3590 from TBLASTN was used for orf generation and further used for reciprocal blast against YALI Lip2.

## Supplementary Information

Supplementary Informations.

## Data Availability

The whole genome sequencing data can be accessed through BioProject Accession Number PRJNA328405. The respective BioSample Accession Numbers is SAMN05170375. The SRA reference numbers of the whole genome sequencing are SRX1850030 (Illumina NextSeq500 short paired-end reads). This Whole Genome sequencing data has been deposited at DDBJ/EMBL/GenBank under the Accession NKYT00000000. The version described in this paper is version NKYT01000000. The datasets generated and analysed during the current study for ITS and D1/D2 rDNA sequences are available at NCBI with Genbank Accession Nos. MK411246 and MK411222 respectively.
