# Supplementary material for: Phylogenomic and biochemical analysis reassesses temperate marine yeast Yarrowia lipolytica NCIM 3590 to be Yarrowia bubula
Source: Sci Rep. 2021 Mar 9;11:5487. doi: 10.1038/s41598-021-83914-6 (PMC7943819; doi:10.1038/s41598-021-83914-6)
Supplement: Supplementary file 1 — Supplementary Informations. [file 41598_2021_83914_MOESM1_ESM.doc]

**Supplementary Information**

**Phylogenomic and biochemical analysis reassesses temperate marine yeast *Yarrowia lipolytica* NCIM 3590 to be *Yarrowia bubula***

Prashant Gaikwad^1#^, Swanand Joshi^1#^, Akshay Mandlecha^1^ & Ameeta RaviKumar^1*^

^1^Microbial Biotechnology Laboratory, Institute of Bioinformatics and Biotechnology, Savitribai Phule Pune University, Ganeshkhind, Pune 411 007, Maharashtra, India

^*^**Corresponding Author**

Prof. Ameeta RaviKumar

E-mail: ameeta@unipune.ac.in

**Supplementary Materials**

**Supplementary Figure 1**

**Supplementary Table S1 - S9**

**Supplementary Materials and Methods**

**2.4. Media and colony morphology of NCIM 3590**

The media component used for studying colony morphology of NCIM 3590

(**a**) Malt extract agar (ME; Malt extract, 5%;), (**b**) Malt extract Glucose Yeast extract Peptone agar (MGYP; Malt extract, 0.3%; Glucose, 1%; Yeast extract, 0.3%; Peptone 0.5%;), (**c**) Potato Dextrose agar (PD; Potato infusion, 20%; Dextrose, 2%;), (**d**) Yeast extract Peptone Glucose agar (YPG; Yeast extract, 0.3%; Peptone, 0.5%; Glucose, 1%;), (**e**) Tributyrin agar (T; Peptone, 0.5%; Yeast extract, 0.3%; Tributyrin, 1%;), (**f**) *Yarrowia lipolytica* Differential Medium (YLDM; Peptone, 0.5%; Yeast extract, 0.5%; L-tyrosine, 0.18%; MnSO_4_ ,0.028%; Lactate, 0.5%;), (**g**) Yeast extract Sucrose agar (YES; KH_2_PO_4_, 0.1%; MgSO_4_, 0.05%; CaCl_2_, 0.05%; NaCl, 0.01%; Yeast extract, 0.05%; Sucrose, 2%;), (**h**) Yeast extract peptone olive oil agar (YPO; Yeast extract, 1%; peptone, 2%; olive oil, 1%;).

**2.8. Lipase activity**

The compositions of the media used for determination of lipase activity

**(a**) Yeast Nitrogen Base containing glucose (YNBG; YNB, 0.7%, glucose, 1%), (**b**) Yeast extract Peptone Glucose; (YPG; Yeast extract, 1%; Peptone, 1%; Glucose, 1%;), (**c**) Yeast extract Peptone Glucose Olive oil (YPGO; Yeast extract, 1%, Peptone, 1%, Glucose, 1% and Olive oil, 1%;), (**d**) Yeast extract Peptone Glucose Tributyrin (YPGTr; Yeast extract, 1%, Peptone, 1%, Glucose, 1%, Tributyrin, 1%;), (**e**) Yeast extract Peptone Glucose Tween 80 (YPGTw; Yeast extract, 1%, Peptone, 1%, Glucose, 1%, Tween 80, 1%).

For Lipase assay, the substrate solution was prepared by adding solution A (30 mg of *p-*NPP in 10 mL of propan-2-ol) to 9.5 mL of solution B (0.1 g of gum arabic and 0.4 g of Triton X-100 in 90 mL of distilled water) drop wise with intense stirring. Triton X-100 was added to clear the substrate suspension. The assay mixture consisted of 180 µL of substrate solution, 20 µL of citrate phosphate buffer (0.5 M, pH 5.0) and 100 µL of extracellular crude enzyme in 96 well microtiter plate. The assay mixture was incubated at 20 °C, 37 °C and 50 °C for 30 min and the *p*-nitro phenol released was measured at 405 nm using Shimadzu UV-1800 (Spectrophotometer, Shimadzu Scientific Instruments Inc., USA). One unit of enzyme activity was expressed as the amount of enzyme that released 1µmol of *p*-nitro phenol per min under the assay conditions. Cell wet weight (g / L) was also determined.

**Results and Discussion**

**ITS and D1/D2 rDNA identification and phylogenetic analysis**

The results suggest that the ITS dataset (314 characters) showed 42 variable sites, 203 conserved sites, 79 parsimony informative sites. On comparisons of the 42 variable sites between *Y. lipolytica* strains and *Y. bubula* strain CBS 12934, species specific base substitutions were seen in 40 sites, with insertions (2) at positions 21 and 294 and a deletion at position (at 5 sites – 16, 23, 50, 285, 286) (Table S2). Similarly, the D1/D2 dataset contained 403 characters, of which 313 conserved sites, 37 variable sites, 70 parsimony-informative. Out of 37 variable sites, 36 were base substitutions and a deletions at position 350 were observed between *Y. lipolytica* and *Y. bubula* strains (Table S3).


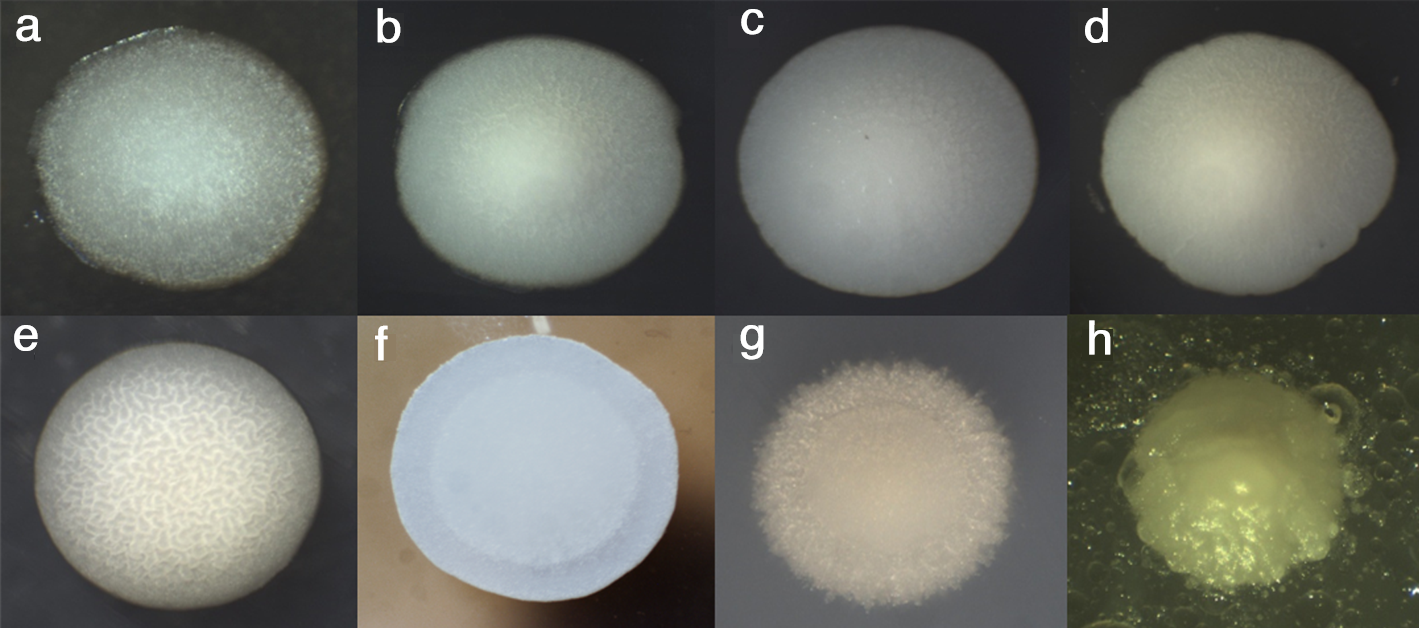


**Fig. S1. Colony morphology of NCIM 3590 grown on different growth media**

(**a**) Malt extract agar (ME), (**b**) Malt extract Glucose Yeast extract Peptone agar (MGYP), (**c**) Potato Dextrose agar (PD), (**d**) Yeast extract Peptone Glucose agar (YPG), (**e**) Tributyrin agar (T), (**f**) *Yarrowia lipolytica* Differential medium (YLDM), (**g**) Yeast extract Sucrose agar (YES) and (**h**) Yeast extract Peptone Olive oil agar (YPO). All the agar plates were incubated at 20 °C for 96 h as mentioned in Materials and Methods.

**Table S1. Isolates used in the phylogenetic analysis**

| **Taxon** | **Culture^a^** | **GenBank Accession** | |
| --- | --- | --- | --- |
|  |  | ITS | D1D2 |
| *Yarrowia lipolytica* | CBS 7504 | AM279249.1 | KY110187.1 |
| *Yarrowia lipolytica* | CBS 7326 | AM279248.1 | KY110190.1 |
| *Yarrowia lipolytica* | CBS 6614 | AM279240.1 | KY110206.1 |
| *Yarrowia lipolytica* | CBS 6317 | AM279238.1 | AM268455.1 |
| *Yarrowia lipolytica* | CBS 6124 | KY105973.1 | KY110181.1 |
| *Yarrowia lipolytica* | CBS 7133 | AM279245.1 | KY110199.1 |
| *Yarrowia lipolytica* | CBS 10143 | KY105989.1 | KY110195.1 |
| *Yarrowia alimentaria* | CBS 10151 | NR_151793.1 | NG_058277.1 |
| *Yarrowia alimentaria* | CBS 10149 | KY101919.1 | KY106283.1 |
| *Yarrowia hollandica* | CBS 4855 | NR_151802.1 | NG_058982.1 |
| *Yarrowia porcina* | CBS 12932 | KY105996.1 | KY110209.1 |
| *Yarrowia porcina* | CBS 12935 | NR_164526.1 | NG_066361 |
| *Yarrowia galli* | CBS 9722 | NR_077078.1 | KY106459.1 |
| *Yarrowia keelungensis* | CBS 11062 | NR_160312.1 | NG_064314.1 |
| *Yarrowia parophonii* | CBS 12427 | KY457248.1 | KY457253.1 |
| *Yarrowia oslonensis* | CBS 10145 | AM279264.1 | AM268476.1 |
| *Yarrowia oslonensis* | CBS10146 | NR_151804.1 | AM26847.1 |
| *Yarrowia divulgata* | CBS 11013 | NR_156000.1 | NG_058444.1 |
| *Yarrowia deformans* | CBS 2076 | KY105959.1 | KY110172.1 |
| *Yarrowia deformans* | CBS 11595 | KY105961.1 | KY110175.1 |
| *Yarrowia deformans* | CBS 10250 | KY105963.1 | KY110174.1 |
| *Yarrowia deformans* | CBS 11593 | KY105960.1 | KY110173.1 |
| *Yarrowia deformans* | CBS 2071 | KY105962.1 | KY110177.1 |
| *Yarrowia bubula* | CBS 12934 | KY105958.1 | NG_059943.1 |
| *Yarrowia lipolytica* | NCIM 3590 | MK411246.1 | MK411222.1 |
| *Yarrowia yakushimensis* | CBS 10253 | KY105999.1 | KY110212.1 |
| *Yarrowia yakushimensis* | CBS 10255 | KY105997.1 | KY110213.1 |
| *Yarrowia yakushimensis* | CBS 10252 | KY106000.1 | KY110210.1 |
| *Yarrowia phangngensis* | CBS 10407 | KY102330.1 | AB304772.1 |

^a^Abbreviations of isolates and culture collections. CBS: Central bureauvoor Schimmel cultures, Utrecht, The Netherlands; NCIM: National Collection of Industrial Microorganisms, Pune.

**Table S2. Variable nucleotide sites with specific ITS base in *Y. lipolytica* and *Y. bubula* groups**

|  | Nucleotide Position | | | | | | | | | | | | | | |
| --- | --- | --- | --- | --- | --- | --- | --- | --- | --- | --- | --- | --- | --- | --- | --- |
| Organism name / Positions | 13 | 16 | 21 | 23 | 28 | 36 | 38 | 40 | 41 | 42 | 43 | 47 | 48 | 50 | 55 |
| *Yarrowia lipolytica* CBS 6124 | T | T | - | C | T | G | T | T | T | C | T | A | T | T | A |
| *Yarrowia lipolytica* CBS 6317 | . | . | - | . | . | . | . | . | . | . | . | . | . | . | . |
| *Yarrowia lipolytica* CBS 6614 | . | . | - | . | . | . | . | . | . | . | . | . | . | . | . |
| *Yarrowia lipolytica* CBS 7133 | . | . | - | . | . | . | . | . | . | . | . | . | . | . | . |
| *Yarrowia lipolytica* CBS 7326 | . | . | - | . | . | . | . | . | . | . | . | . | . | . | . |
| *Yarrowia lipolytica* CBS 7504 | . | . | - | . | . | . | . | . | . | . | . | . | . | . | . |
| *Yarrowia lipolytica* CBS 10143 | . | . | - | . | . | . | . | . | . | . | . | C | . | . | . |
| *Yarrowia bubula* CBS 12934 | A | - | C | - | G | A | A | A | C | T | A | . | A | - | C |
| *Yarrowia lipolytica* NCIM 3590* | A | - | C | - | G | A | A | A | C | T | A | . | A | - | C |
|  | Nucleotide Position | | | | | | | | | | | | | |  |
| Organism name / Positions | 56 | 57 | 58 | 62 | 63 | 64 | 65 | 69 | 192 | 203 | 210 | 218 | 244 | 264 | 265 |
| *Yarrowia lipolytica* CBS 6124 | G | C | G | T | T | T | T | T | A | T | C | G | T | T | T |
| *Yarrowia lipolytica* CBS 6317 | . | . | . | . | . | . | . | . | . | . | . | . | . | . | . |
| *Yarrowia lipolytica* CBS 6614 | . | . | . | . | . | . | . | . | . | . | . | . | . | . | . |
| *Yarrowia lipolytica* CBS 7133 | . | . | . | . | . | . | . | . | . | . | . | . | . | . | . |
| *Yarrowia lipolytica* CBS 7326 | . | . | . | . | . | . | . | . | . | . | . | . | . | . | . |
| *Yarrowia lipolytica* CBS 7504 | . | . | . | . | . | . | . | . | . | . | . | . | . | . | . |
| *Yarrowia lipolytica* CBS 10143 | . | . | . | . | . | . | . | . | . | . | . | . | . | . | . |
| *Yarrowia bubula* CBS 12934 | T | A | A | A | A | A | C | C | G | C | T | A | C | G | A |
| *Yarrowia lipolytica* NCIM 3590* | T | A | A | A | A | A | C | C | G | C | T | A | C | G | A |
|  | Nucleotide Position | | | | | | | | | | | |  |  |  |
| Organism name / Positions | 274 | 276 | 277 | 281 | 282 | 284 | 285 | 286 | 288 | 294 | 302 | 310 |  |  |  |
| *Yarrowia lipolytica* CBS 6124 | C | C | A | A | T | A | C | G | C | - | C | G |  |  |  |
| *Yarrowia lipolytica* CBS 6317 | . | . | . | . | . | . | . | . | . | - | . | . |  |  |  |
| *Yarrowia lipolytica* CBS 6614 | . | . | . | . | . | . | . | . | . | - | . | . |  |  |  |
| *Yarrowia lipolytica* CBS 7133 | . | . | . | . | . | . | . | . | . | - | . | . |  |  |  |
| *Yarrowia lipolytica* CBS 7326 | . | . | . | . | . | . | . | . | . | - | . | . |  |  |  |
| *Yarrowia lipolytica* CBS 7504 | . | . | . | . | . | . | . | . | . | - | . | . |  |  |  |
| *Yarrowia lipolytica* CBS 10143 | . | . | . | . | . | . | . | . | . | - | . | . |  |  |  |
| *Yarrowia bubula* CBS 12934 | A | T | C | T | A | C | - | - | A | C | T | A |  |  |  |
| *Yarrowia lipolytica* NCIM 3590* | A | T | C | T | A | C | - | - | A | C | T | A |  |  |  |

**Table S3. Variable nucleotide sites with specific D1/D2 base in *Y. lipolytica* and *Y. bubula* groups**

|  | Nucleotide Position | | | | | | | | | | | | | | |
| --- | --- | --- | --- | --- | --- | --- | --- | --- | --- | --- | --- | --- | --- | --- | --- |
| Organism name / Positions | 56 | 61 | 62 | 63 | 80 | 130 | 131 | 132 | 141 | 311 | 318 | 328 | 329 | 330 | 331 |
| *Yarrowia lipolytica* CBS 6124 | C | G | A | G | G | C | T | C | G | A | T | C | C | G | A |
| *Yarrowia lipolytica* CBS 7504 | . | . | . | . | . | . | . | . | . | . | . | . | . | . | . |
| *Yarrowia lipolytica* CBS 7326 | . | . | . | . | . | . | . | . | . | . | . | . | . | . | . |
| *Yarrowia lipolytica* CBS 6614 | . | . | . | . | . | . | . | . | . | . | . | . | . | . | . |
| *Yarrowia lipolytica* CBS 6317 | . | . | . | . | . | . | . | . | . | . | . | . | . | . | . |
| *Yarrowia lipolytica* CBS 7133 | . | . | . | . | . | . | . | . | . | . | . | . | . | . | . |
| *Yarrowia lipolytica* CBS 10143 | . | . | . | . | . | . | . | . | . | . | . | . | . | . | . |
| *Yarrowia bubula* CBS 12934 | A | A | G | A | A | T | C | T | T | G | C | T | G | A | T |
| *Yarrowia lipolytica* NCIM 3590* | A | A | G | A | A | T | C | T | T | G | C | T | G | A | T |
|  | Nucleotide Position | | | | | | | | | | | | | |  |
| Organism name / Positions | 332 | 341 | 345 | 346 | 350 | 351 | 355 | 361 | 365 | 366 | 367 | 368 | 379 | 381 | 382 |
| *Yarrowia lipolytica* CBS 6124 | G | C | C | C | T | G | G | C | C | G | A | C | C | A | T |
| *Yarrowia lipolytica* CBS 7504 | . | . | . | . | . | . | . | . | . | . | . | . | . | . | . |
| *Yarrowia lipolytica* CBS 7326 | . | . | . | . | . | . | . | . | . | . | . | . | . | . | . |
| *Yarrowia lipolytica* CBS 6614 | . | . | . | . | . | . | . | . | . | . | . | . | . | . | . |
| *Yarrowia lipolytica* CBS 6317 | . | . | . | . | . | . | . | . | . | . | . | . | . | . | . |
| *Yarrowia lipolytica* CBS 7133 | . | . | . | . | . | . | . | . | . | . | . | . | . | . | . |
| *Yarrowia lipolytica* CBS 10143 | . | . | . | . | . | . | . | . | . | . | . | . | . | . | . |
| *Yarrowia bubula* CBS 12934 | A | G | G | T | - | C | C | T | T | T | T | A | G | T | G |
| *Yarrowia lipolytica* NCIM 3590* | A | G | G | T | - | C | C | T | T | T | T | A | G | T | G |
|  | Nucleotide Position | | | | | | | |  |  |  |  |  |  |  |
| Organism name / Positions | 385 | 387 | 388 | 389 | 391 | 397 | 400 |  |  |  |  |  |  |  |  |
| *Yarrowia lipolytica* CBS 6124 | C | A | G | A | C | T | C |  |  |  |  |  |  |  |  |
| *Yarrowia lipolytica* CBS 7504 | . | . | . | . | . | . | . |  |  |  |  |  |  |  |  |
| *Yarrowia lipolytica* CBS 7326 | . | . | . | . | . | . | . |  |  |  |  |  |  |  |  |
| *Yarrowia lipolytica* CBS 6614 | . | . | . | . | . | . | . |  |  |  |  |  |  |  |  |
| *Yarrowia lipolytica* CBS 6317 | . | . | . | . | . | . | . |  |  |  |  |  |  |  |  |
| *Yarrowia lipolytica* CBS 7133 | . | . | . | . | . | . | . |  |  |  |  |  |  |  |  |
| *Yarrowia lipolytica* CBS 10143 | . | . | . | . | . | . | . |  |  |  |  |  |  |  |  |
| *Yarrowia bubula* CBS 12934 | T | G | A | G | A | A | T |  |  |  |  |  |  |  |  |
| *Yarrowia lipolytica* NCIM 3590* | T | G | A | G | A | A | T |  |  |  |  |  |  |  |  |

**Table S4. Average percent divergence of NCIM 3590 strain with *Y. lipolytica* and *Y. bubula* groups using *p*-distance method ^a^**

| *Yarrowia* Group / Clade | NCIM 3590 | |
| --- | --- | --- |
|  | Divergence (%) | |
|  | ITS | D1/D2 |
| *Y. bubula* | 0.00 | 0.00 |
| *Y. lipolytica* | 13.35 | 9.19 |
| *Y. hollandica* | 9.79 | 7.73 |
| *Y. porcina* | 11.64 | 10.47 |
| *Y. deformans* | 11.36 | 9.78 |
| *Y.divulgata* | 11.30 | 9.22 |
| *Y. parophonni* | 12.62 | 9.22 |
| *Y. keelungensis* | 13.74 | 9.97 |
| *Y. alimentaria* | 13.90 | 11.09 |
| *Y. oslonensis* | 13.60 | 9.47 |
| *Y. galli* | 12.15 | 9.72 |
| *Y. yakushimensis* | 14.56 | 10.22 |
| *Y. phangngensis* | 16.66 | 11.47 |

^a^Evolutionary analyses were conducted in MEGA X as mentioned in materials and methods. The number of base differences per site from averaging over all sequence pairs between groups are shown. This analysis involved 30 nucleotide sequences. There were a total of 405 positions for D1/D2 and 314 positions for ITS in the final dataset. *Y. bubula* group contains one strain while *Y. lipolytica* group with 7 strains

**Table S5. Comparative statistics of NCIM 3590 with reference *Yarrowia* strains ^a^.**

| **Sr. No** | **Parameters** | **NCIM 3590** | **CBS 12934** | **CLIB 122** | **CLIB 89** |
| --- | --- | --- | --- | --- | --- |
| 01 | Strain | NCIM 3590 | CBS 12934 | CLIB 122 | CLIB 89 (W29) |
| 02 | BioSample | SAMN05170375 | SAMNEA4824134 | SAMEA3138171 | SAMN04088558 |
| 03 | Bioproject | PRJNA328405 | PRJEB18081 | PRJNA13837 | PRJNA295780 |
| 04 | Assembly | GCA_003571375.1 | GCA_900519075.1 | GCA_000002525.1 | GCA_001761485.1 |
| 05 | Level | Scaffold/Super Scaffold | Scaffold | Chromosome | Complete |
| 06 | Contigs | 2,485 | NA | 19 | 7 |
| 07 | Contig N50 | 45,970 | 1,48,662 | 2,069,334 | NA |
| 08 | Contig L50 | 123 | 46 | 4 | NA |
| 09 | Scaffolds | 485 | 28 | 7 | 7 |
| 10 | Scaffold N50 | 73,209 | 1,641,727 | 3,633,272 | 3,629,463 |
| 11 | Scaffold L50 | 77 | 4 | 4 | 3 |
| 12 | Genome size (Mb) | 20.02 | 20.90 | 20.55 | 20.55 |
| 13 | GC content (%) | 47.00 | 46.20 | 48.98 | 48.98 |

^a^ data obtained for NCIM 3590 was compared with CBS 12934, CLIB 122 and CLIB 89 from NCBI database

**Table S6. Characteristics of colony morphologies by NCIM 3590 on different solid media.**

| Sr.No | Medium^*^ | Colony | Form | Elevation | Margin | Edge | Surface | Opacity | Chromogenesis |
| --- | --- | --- | --- | --- | --- | --- | --- | --- | --- |
| **a** | ME | Circular | Circular | Umbonate | Entire | Entire | Wrinkled | Opaque | White |
| **b** | MGYP | Circular | Circular | Umbonate | Entire | Entire | Wrinkled | Opaque | White |
| **c** | PDA | Circular | Circular | Umbonate | Entire | Entire | Wrinkled | Opaque | White |
| **d** | YPG | Circular | Circular | Umbonate | Entire | Entire | Wrinkled | Opaque | White |
| **e** | T | Circular | Circular | Umbonate | Entire | Entire | Wrinkled | Opaque | White |
| **f** | YLDM | Circular | Circular | Umbonate | Entire | Entire | Wrinkled | Opaque | White |
| **g** | YES | Circular | Circular | Umbonate | Erose | Filamentous | Wrinkled | Opaque | White |
| **h** | YPO | Circular | Circular | Raised | Undulate | Undulate | Dry/Powdery | Opaque | Yellow |

**^*^a**. Malt extract agar (ME), **b**. Malt extract glucose yeast extract peptone agar (MGYP), **c**. Potato dextrose agar (PD), **d**. Yeast extract peptone glucose agar (YPG), **e**. Tributyrin agar (T), **f**. *Yarrowia lipolytica* Differential medium (YLDM), **g**.Yeast extract sucrose agar (YES) and **h**. Yeast extract peptone Olive oil agar (YPO). All the agar plates were incubated at 20 °C for 96 h.

**Table S7. Sugar assimilation and oxidation tests of NCIM 3590.**

| **Sr. no.** | **Carbon Source ^a^** | **NCIM 3590** |  | **Sr. no.** | **Carbon Source ^a^** | **NCIM 3590** |  | **Sr. no.** | **Substrates ^b^** | **NCIM 3590** |
| --- | --- | --- | --- | --- | --- | --- | --- | --- | --- | --- |
| 1 | Water | - |  | 31 | L-sorbose | + |  | 1 | Water | - |
| 2 | Fumaric acid | ++ |  | 32 | α-Methyl D-glucoside | - |  | 2 | Acetic acid | - |
| 3 | L-Malic acid | ++ |  | 33 | β-Methyl D-glucoside | + |  | 3 | Formic acid | - |
| 4 | Methyl succinate | + |  | 34 | Amygdalin | + |  | 4 | Propionic acid | - |
| 5 | Bromosuccinc acid | + |  | 35 | Arbitun | - |  | 5 | Succinic acid | + |
| 6 | L-glutamic acid | ++ |  | 36 | Salicin | + |  | 6 | Methyl succinate | - |
| 7 | γ-amino- butyric acid | ++ |  | 37 | Maltitol | - |  | 7 | L-Aspartic acid | + |
| 8 | α-keto-glutaric acid | + |  | 38 | D-Mannitol | + |  | 8 | L-Aspartic acid | ++ |
| 9 | 2-keto-D-gluconic | ++ |  | 39 | D-Sorbitol | + |  | 9 | L-Proline | ++ |
| 10 | D-gluconic acid | ++ |  | 40 | Adonitol | + |  | 10 | D-Gluconic acid | + |
| 11 | Dextrin | - |  | 41 | D-Arabitol | + |  | 11 | Dextrin | - |
| 12 | Inulin | - |  | 42 | Xylitol | + |  | 12 | Inulin | - |
| 13 | D-Cellobiose | - |  | 43 | i-Erythritol | ++ |  | 13 | D-Cellobiose | - |
| 14 | Gentiobiose | + |  | 44 | Glycerol | + |  | 14 | Gentiobiose | - |
| 15 | Maltose | - |  | 45 | Tween 80 | + |  | 15 | Maltose | - |
| 16 | Maltotriose | - |  | 46 | L-Arabinose | + |  | 16 | Maltotriose | - |
| 17 | D-Melezitose | - |  | 47 | D-Arabinose | + |  | 17 | D-Melezitose | - |
| 18 | D-Melibiose | - |  | 48 | D-Ribose | + |  | 18 | D-Melibiose | - |
| 19 | Palatinose | - |  | 49 | D-Xylose | + |  | 19 | Palatinose | - |
| 20 | D-raffinose | - |  | 50 | Methyl succinate +D-xylose | + |  | 20 | D-Raffinose | - |
| 21 | Stachyose | - |  | 51 | N-acetyl-L-glutamic acid + D-xylose | + |  | 21 | Stachyose | - |
| 22 | Sucrose | - |  | 52 | Quinic acid + D-xylose | + |  | 22 | Sucrose | - |
| 23 | D-Trehalose | - |  | 53 | D-glucuronic acid + D-xylose | - |  | 23 | D-Trehalose | - |
| 24 | Turanose | - |  | 54 | Dextrin +D-xylose | + |  | 24 | Turanose | - |
| 2514 | *N*-acetyl-D-glucosamine | + |  | 55 | a-D-lactose+D-xylose | + |  | 25 | *N*-acetyl-D-glucosamine | + |
| 26 | D-Glucosamine | - |  | 56 | D-melibiose+D-xylose | + |  | 26 | α-D-glucose | + |
| 27 | α-D-glucose | ++ |  | 57 | D-Galactose+D-xylose | - |  | 27 | D-Galactose | - |
| 28 | D-Galactose | + |  | 58 | m-inositol+D-Xylose | + |  | 28 | D-Psicose | - |
| 29 | D-Psicose | - |  | 59 | 1,2-propanediol+D-xylose | + |  | 29 | L-Sorbose | - |
| 30 | L-Rhamnose | - |  | 60 | Acetoin+D-xylose | - |  | 30 | Salicin | + |
|  |  |  |  |  |  |  |  | 31 | D-Mannitol | + |
|  |  |  |  |  |  |  |  | 32 | D-Sorbitol | + |
|  |  |  |  |  |  |  |  | 33 | D-Arabitol | + |
|  |  |  |  |  |  |  |  | 34 | Xylitol | - |
|  |  |  |  |  |  |  |  | 35 | Glycerol | + |
|  |  |  |  |  |  |  |  | 36 | Tween 80 | - |

^b^Substrates used for oxidation. The yeast cells were grown at 20^0^C for 72 h. (++) indicates strong assimilation, (+) weak assimilation and (-) no assimilation.

**Table S8. Effect of different media on extracellular lipase production by NCIM 3590.**

| Sr. No | Media# | Extracellular lipase activity | Wet Cell mass |
| --- | --- | --- | --- |
|  |  | U / mL | g / L |
| 1 | YNB | ND | 396 |
| 2 | YPG | 0.008 | 489 |
| 3 | YPGO | ND | 532 |
| 4 | YPGTr | 0.003 | 181 |
| 5 | YPGTw | ND | 500 |

^#^The culture was grown in different media and incubated for 72 h at 20 °C with shaking at 120 rpm. The values given in the table are the average of two independent experiments. ^ND^ – not detected. One unit of enzyme activity was expressed as the amount of enzyme that released 1 μmol of *p*-nitrophenol per min under the assay conditions.

**Table S9. BLAST analysis of *LIP2* gene with different *Yarrowia* genomes.**

| **Sr.**  **no** | **Organism Name** | **Sequence Id** | **Contig** | **Alignment length** | **Identical** | **% Identity** | **Positives** | **Query Coverage** | **E Value** |
| --- | --- | --- | --- | --- | --- | --- | --- | --- | --- |
| 1 | *Y. lipolytica* CLIB122 | CR382127.1 | Chromosome A | 334 | 334 | 100 | 334 | 100 | 0 |
| 2 | *Y. lipolytica* CLIB89 | CP017553.1 | Chromosome1A | 334 | 334 | 100 | 334 | 100 | 0 |
| 3 | *Y. galli* CBS 9722 | ULGS01000005.1 | YAGA0E | 334 | 309 | 93 | 325 | 97 | 0 |
| 4 | *Y. divulgata* CBS 11013 | ULGQ01000001.1 | YADI0S01 | 334 | 311 | 93 | 323 | 97 | 0 |
| 5 | *Y. deformans* CBS 2071 | ULGY01000018.1 | YADE0S18 | 334 | 306 | 92 | 324 | 97 | 0 |
| 6 | *Y. osloensis* CBS 10146 | ULGU01000007.1 | YAOS0S07 | 334 | 304 | 91 | 323 | 97 | 0 |
| 7 | *Y. hollandica* CBS 4855 | ULGV01000004.1 | YAHO0S04 | 316 | 285 | 90 | 299 | 95 | 0 |
| 8 | *Y alimentaria* CBS 10151 | ULGN01000010.1 | YAAL0S10 | 334 | 293 | 88 | 311 | 93 | 0 |
| 9 | *Y. keelungensis* CBS 11062 | ULGP01000009.1 | YAKE0S09 | 334 | 288 | 86 | 321 | 96 | 0 |
| 10 | *Y. bubula* CBS 12934 | ULGX01000001.1 | YABU0S01 | 313 | 274 | 88 | 297 | 95 | 0 |
| 11 | *Y. yakushimensis* CBS 10253 | ULGW01000006.1 | YAYA0S6 | 313 | 268 | 86 | 294 | 94 | 0 |
| 12 | *Y. porcina* CBS 12935 | ULGT01000017.1 | YAPO0S17 | 288 | 270 | 94 | 280 | 97 | 0 |
| 13 | *Y. phangngaensis* CBS 10407 | ULGR01000006.1 | YAPH0S6 | 316 | 259 | 82 | 291 | 92 | 0 |
| 14 | *Y. lipolytica* NCIM 3590 | NKYT01000426.1 | scaffold57 | 304 | 129 | 42 | 183 | 60 | 2e-77 |
